# *Mycoplasma hominis* Induces Mediastinitis after a Tonsillar Abscess

**DOI:** 10.1155/2016/9531715

**Published:** 2016-11-10

**Authors:** Anna Grancini, Manuela Colosimo, Antonella Restelli, Rosaria Colombo, Anna Maraschini, Cristina Pozzi, Giuseppe Breda, Alessandro Protti, Milena Arghittu, Luca Gallelli, Rita Maiavacca

**Affiliations:** ^1^Clinical Chemistry and Microbiology Central Laboratory, Milan, Italy; ^2^ITU E. Vecla, Fondazione IRCCS “Cà Granda” Ospedale Maggiore Policlinico, Milano, Italy; ^3^Clinical Pharmacology and Pharmacovigilance Unit, AO Mater Domini, Department of Health Science, University of Catanzaro, Catanzaro, Italy

## Abstract

*Mycoplasma hominis* is commonly involved in genitourinary tract infections. We report a 59-year-old man who developed a* M. hominis*-associated mediastinitis following acute tonsillar infection.

## 1. Background

*Mycoplasma hominis *is a bacterium frequently isolated from urogenital and respiratory tracts of asymptomatic healthy individuals [1]. In patients with risk factors for infections, for example, immunosuppression, trauma, or poor cardiorespiratory function, it may induce systemic infections [2]. In this paper we report the development of mediastinitis due to* M. hominis *following tonsillar abscess.

## 2. Presentation of the Case

A 59-year-old man with septic shock and purulent mediastinitis was admitted to our observation on June 1, 2015, to undergo thoracotomy. History revealed that on May 24, 2015, for a tonsillar abscess he was hospitalized in the Otolaryngology Unit in another Italian city, and an empiric antimicrobial treatment was started.

Four days later (May 28), for a septic shock he was admitted to the Intensive Care Unit (ICU) of the same hospital where a computer tomography (CT) revealed the presence of pleural emphysema and right pulmonary embolism, while a culture of pus, taken at the time of the admission, showed an infection sustained by the group F of* Streptococcus beta-haemolyticus*. A treatment with amoxicillin and enoxaparin was started; a thoracic drainage was also positioned with an initial improvement of symptoms (body temperature 37.3°C, blood pressure 108/65 mmHg). On June 1, clinical conditions acutely impaired (body temperature 39°C, blood pressure 100/60 mmHg), while the CT of head and thorax documented a large abscess from the left tonsil to the anterior mediastinum and retropharyngeal and paracardiac regions up to right lung where an emphysema was developed. No radiological signs of pulmonary embolism were showed. Physicians performed the drainage of the retropharyngeal abscess but due to the impossibility to treat the thorax abscess, on June 2, the patient was transferred to the ICU of our hospital.

At the admission (June 2), the patient was unconscious with a body temperature of 39°C, blood pressure was 100/60 mmHg, and heart frequency was 105 bits/min arrhythmic, while respiratory rate was 55/min; electrocardiography excluded the presence of an ischemic heart disease but documented a supraventricular fibrillation; therefore, amiodarone was quickly started. Biochemical findings showed an increase in creatinine plasma levels (1.8 mg/dL; CKD-EPI: Chronic Kidney Disease Epidemiology Collaboration: 53), C-reactive protein (CRP; 32 mg/dL), erythrocyte sedimentation rate (ESR 125 mm/h), procalcitonin (PCT 9 ng/mL), and white blood cells (16,900 per *μ*L) and a decrease in blood platelets values (84,000/mL), supporting the diagnosis of septic shock. The patient underwent emergency thoracotomy with a left enlarged cervicotomy, a debridement of the infected tissues, and drainage of the mediastinum. Both abscess biopsies and samples of pus were sent to the bacteriological laboratory for a microbiological evaluation. At this time a treatment with nadroparin (0.6 mL), amoxicillin + clavulanic acid (1 gr/8 h), and pantoprazole (20 mg) as well as parental nutrition was started.

On June 7, microbiology tests revealed the presence of anaerobic culture with pinpoint colonies in the blood agar Schaedler plates.

On June 9 and 18, the patient underwent two thorax surgical revisions and on 18 June amoxicillin + clavulanic acid was stopped and replaced with meropenem and linezolid. On June 22, mass spectrometry (Maldi-Tof MS bioMerieux) revealed in cultures obtained on June 1, 9, and 18 the presence of* M. hominis*, confirmed by MycoView (Zeakon Diagnostics, France).

Antibiogram showed that the strain was sensitive to tetracycline (MIC 0.125 *μ*g/mL) and clindamycin (MIC 0.06 *μ*g/mL) and resistant to quinolones and macrolides (MIC 4 and 8 *μ*g/mL, resp.). Meropenem and linezolid were discontinued in favor of doxycycline with a rapid clinical improvement of symptoms (FR 20, BP 120/80 mm/Hg, and heart frequency 69 b/m) in about 7 days; after this time doxycycline was stopped and the patient made a complete improvement in about 4 months. At this time CT did not show evidence of thoracic infection and no signs of infection were recorded during the examination.

## 3. Discussion

We present a case of mediastinitis due to* M. hominis* after a tonsillar abscess. To date, in literature have been reported 17 cases of mediastinitis induced by* M. hominis* infection in men after organ transplant (heart or lung), heart surgery, or immunosuppressive therapy [3–9]. Symptoms, that is, pain and hyperthermia, usually appear within 14 days from the surgery. However, Kennedy et al. reported a case of* M. hominis*-associated parapharyngeal abscess following acute Epstein-Barr virus infection in a previously immunocompetent young patient without history of surgery or trauma [10]. In the present case we report an immunocompetent 59-year-old man without a history of surgery but with a clinical diagnosis of tonsillar abscess complicated with septic shock. Unfortunately the diagnosis was performed about 20 days after the admission due to the germ characteristics. In fact, mycoplasma is a small and gram-negative bacterium with a slow growth and with no cell wall, which means that it requires a specialized medium not routinely used. Moreover, the inability to detect growth of these organisms using automated blood cultures is attributed to the mycoplasmastatic effects of sodium polyanethol sulfonate, the anticoagulant widely used in the liquid blood cultures media, although this problem can be overcome by the addition of gelatine [11]. However,* M. hominis* only may be detected in the routine bacteriological media (such as blood agar; from 2 to 4 days, usually), typically as pinpoint translucent colonies with a fried egg morphology [12], even if this method of isolation is not always reliable. In our case, the germ's characteristic explains the inefficacy of the empiric treatment and it probably induces the progression of the disease delaying the diagnosis. Both surgical drainage and debridement are the keys to recovery and these probably reduced the progression of the infection and improved the symptoms, and it is also difficult to determine whether the clinical response was solely due to surgical drainage or a surgery and antibiotics mixture. The state of the immunocompetent patient has certainly allowed the good resolution of this case, even without the support of a specific antibiotic therapy.

In this context, it is also important to have a good microbiological evaluation in patients with a tonsillar abscess in order to exclude* M. hominis* infection/coinfection. This is why only a quick diagnosis and a correct treatment may reduce the risk of a systemic infection decreasing the costs of hospitalization.

Finally, we report for the first time the development of mediastinitis inducted by* M. hominis* in an immunocompetent patient with a tonsillar abscess; however, it is important to underline that this is a case report and other data are necessary to validate our observation.

## References

[1] Mattila P. S., Carlson P., Sivonen A. (1999). Life-threatening Mycoplasma hominis mediastinitis. *Clinical Infectious Diseases*.

[2] Miranda C., Camacho E., Reina G. (2005). Isolation of Mycoplasma hominis from extragenital cultures. *European Journal of Clinical Microbiology and Infectious Diseases*.

[3] Sielaff T. D., Everett J. E., Shumway S. J., Wahoff D. C., Bolman R. M., Dunn D. L. (1996). Mycoplasma hominis infections occurring in cardiovascular surgical patients. *Annals of Thoracic Surgery*.

[4] Hopkins P. M., Winlaw D. S., Chhajed P. N. (2002). Mycoplasma hominis infection in heart and lung transplantation. *Journal of Heart and Lung Transplantation*.

[5] García-de-la-Fuente C., Miñambres E., Ugalde E., Sáez A., Martinez-Martinez L., Fariñas M. C. (2008). Post-operative mediastinitis, pleuritis and pericarditis due to *Mycoplasma hominis* and *Ureaplasma urealyticum* with a fatal outcome. *Journal of Medical Microbiology*.

[6] Myers P. O., Khabiri E., Greub G., Kalangos A. (2010). Mycoplasma hominis mediastinitis after acute aortic dissection repair. *Interactive Cardiovascular and Thoracic Surgery*.

[7] Karaca S., Kalangos A. (2015). Vacuum-assisted closure (VAC)-Instill with continuous irrigation for the treatment of Mycoplasma hominis mediastinitis. *International Wound Journal*.

[8] Le Guern R., Loïez C., Loobuyck V., Rousse N., Courcol R., Wallet F. (2015). A new case of Mycoplasma hominis mediastinitis and sternal osteitis after cardiac surgery. *International Journal of Infectious Diseases*.

[9] Boyle E. M., Burdine J., Bolman R. M. (1993). Successful treatment of Mycoplasma mediastinitis after heart-lung transplantation. *Journal of Heart and Lung Transplantation*.

[10] Kennedy K. J., Prince S., Makeham T. (2009). Mycoplasma hominis-associated parapharyngeal abscess following acute Epstein-Barr virus infection in a previously immunocompetent adult. *Journal of Clinical Microbiology*.

[11] Waites K. B., Canupp K. C. (2001). Evaluation of BacT/ALERT system for detection of Mycoplasma hominis in simulated blood cultures. *Journal of Clinical Microbiology*.

[12] Kitson K. A., Wright K. C. (1996). Isolation of Mycoplasma hominis using the BACTEC 9000 series blood culture system. *Journal of Clinical Pathology*.

